# Malignant Syphilis in a Female Patient: A Case Report and Mini-Review

**DOI:** 10.3390/tropicalmed7030047

**Published:** 2022-03-08

**Authors:** Julija Dimnik, Maja Benko, Violeta Hosta, Andreja Murnik Rauh, Andreja Pagon, Vesna Cvitković Špik, Saba Battelino, Domen Vozel

**Affiliations:** 1 Department of Dermatovenereology, University Medical Centre Ljubljana, SI-1000 Ljubljana, Slovenia; julija.dimnik@kclj.si (J.D.); maja.benko@kclj.si (M.B.); violeta.hosta@kclj.si (V.H.); andreja.murnik@kclj.si (A.M.R.); andrejka.pagon@gmail.com (A.P.); 2 Faculty of Medicine, Institute of Microbiology and Immunology, University of Ljubljana, SI-1000 Ljubljana, Slovenia; vesna.cvitkovic-spik@mf.uni-lj.si; 3 Department of Otorhinolaryngology and Cervicofacial Surgery, University Medical Centre Ljubljana, SI-1000 Ljubljana, Slovenia; saba.battelino@kclj.si; 4 Faculty of Medicine, University of Ljubljana, SI-1000 Ljubljana, Slovenia

**Keywords:** sexually transmitted diseases, syphilis serodiagnosis, otolaryngology, dermatology, diagnostic errors

## Abstract

Malignant syphilis (MS) is a rare form of secondary syphilis with grotesque skin lesions, systemic manifestation and life-threatening complications. This article presents a case of MS in an immunocompetent 41-year-old female, who initially manifested with a generalized nonpruritic erythematous rash and systemic symptoms. She was mistreated for generalized impetigo and hepatitis attributed to chronic alcoholism. After partial recovery and a 3-month latent period, she developed infiltrated plaques with crusts on the trunk, head and neck; pharyngitis and laryngeal lesions; generalized lymphadenopathy and nonspecific systemic symptoms. Serologic tests confirmed syphilis, and cerebrospinal fluid analyses indicated the presence of anti-treponemal antibodies. Urine drug screening was positive for cannabinoids. The polymerase chain reaction from skin biopsy samples identified *T. pallidum*, confirmed with Warthin-Starry staining. Immunohistochemical analysis was uncharacteristic. Tertiary syphilis, neurosyphilis, ocular syphilis and otosyphilis were excluded. However, the patient was treated for neurosyphilis with benzylpenicillin (18 million IU intravenously daily, 14 days) and corticosteroids. No Jarisch-Herxheimer reaction occurred. Ten months after treatment, residual scars were visible, and 1 year later, she attempted suicide. Since MS can resemble other diseases, it should be suspected in a mentally ill patient with chronic drug abuse, systemic nonspecific manifestations and dermatological abnormalities, including the head and neck region.

## 1. Introduction

Malignant syphilis (MS), also called nodulo-ulcerative syphilis, is a rare infectious disease first described by Bazin in 1859 [1]. It is classified as a form of secondary syphilis, which typically occurs due to immunosuppression [2], typically from 6 weeks to 1 year after the first manifestations of primary syphilis [3]. A German study showed that 7.3% of participating human immunodeficiency virus- (HIV-) positive patients were affected by MS [4]. Nevertheless, some reports of patients with MS suffered from malnutrition, chronic alcohol abuse, hepatitis, pregnancy, diabetes or other debilitating diseases that also cause immune deficiency [5].

This article describes an interesting case of MS in a female patient without known immune deficiencies, which are classically present in MS. The role of the immune system and the transmission mode is questioned in this patient and MS.

The case is presented according to CARE guidelines [6].

## 2. Case Presentation

### 2.1. History

A 41-year-old female was admitted to the Department of Dermatovenereology, University Medical Centre Ljubljana, Slovenia, in December 2019 due to thick crusts and a rash on her face, scalp, neck, trunk and extremities.

Approximately 3 months before admission, she noted a nonpruritic erythematous rash on her trunk and arms that spread to her face. When the first lesions appeared, she received oral therapy with 500 mg azithromycin for 3 consecutive days and topical antibiotic therapy. She was also examined at the same dermatovenereology outpatient service. Impetigo was suspected. A skin swab for pathogenic bacteria and a native mycological examination were performed, and both gave negative results. A biopsy was advised for histopathological evaluation and cultivation for atypical mycobacteria, but the patient did not perform it against advice.

Meanwhile, she reported malaise, loss of appetite, abdominal pain, vomiting, hair loss and chills; for that reason, she was briefly admitted to the Internal Medicine Department. Due to elevated values of liver enzymes and carbohydrate-deficient transferrin (CDT) and diffuse parenchymal liver damage identified on abdominal ultrasound, the patient was diagnosed with chronic alcoholic hepatitis. 

Otherwise, the patient was healthy and was taking no regular medication. She reported a lobectomy of the left lung due to a benign lesion and had had an appendectomy at 13. She was an occasional alcohol user and a marijuana smoker. She had unconfirmed allergies to penicillin (history of angioedema), clindamycin, thiethylperazine, clarithromycin, diclofenac, ketoprofen and iodinated contrast. Family history was positive for psoriasis and arterial hypertension. In the past year, she had had two male sexual partners. She was an unemployed registered nurse.

### 2.2. Investigations and Differential Diagnosis

#### 2.2.1. General Physical Examination

**Figure 1:**
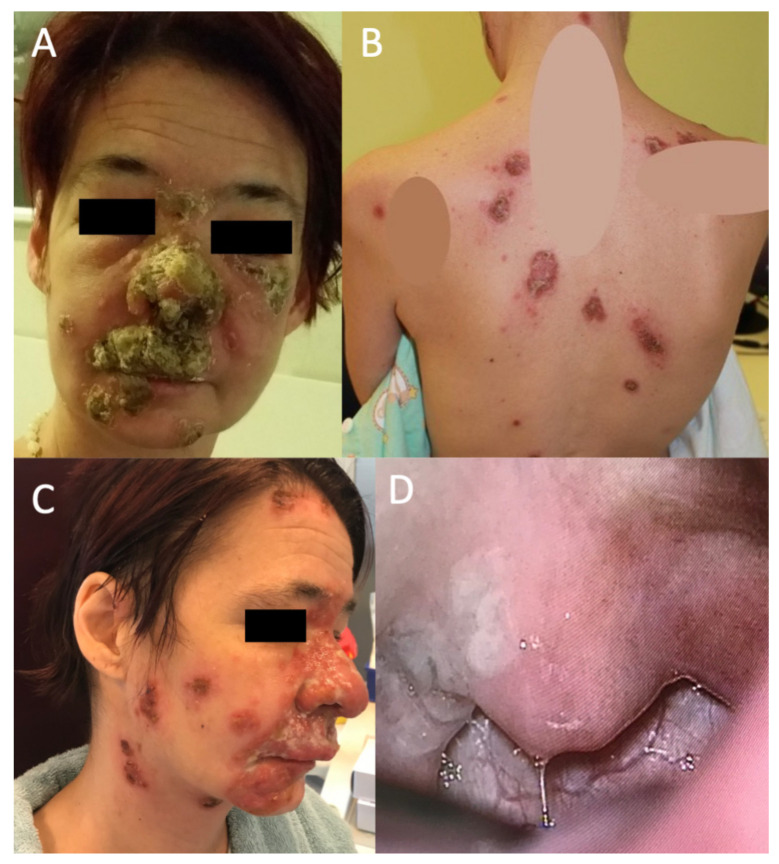
Female patient with malignant syphilis. (**A**) Extensive plaques with crusts and erosions on her face at initial presentation. (**B**) Annular lesions with crusts on the torso (tattoos are concealed). (**C**) Extensive plaques, ulcers and erosions. Crusts were removed with ointment treatment. Diffuse swelling of the nasal soft tissues is visible. (**D**) Right-sided tonsillar plaque, consistent with syphilitic angina. The posterior oropharyngeal wall is hyperemic (*N.B.* enhanced vascular pattern).

There were infiltrated plaques with crusts and erosions on her nose, cheeks, neck, scalp, trunk and extremities (Figure 1). In addition, small, nontender and freely movable supraclavicular and inguinal lymph nodes were palpable. She was in no acute distress, was afebrile and normally hydrated, but she was malnourished. Later, she became febrile up to 38.5 °C and complained of a constant sore throat during hospitalization.

#### 2.2.2. Laboratory Test Results

Serologic tests showed a positive rapid plasma regain (RPR; RPR Card test, Becton, Dickinson and Company, Sparks, MD, USA) at a titer of 1:128, as well as *T. pallidum* particle agglutination assay (TPPA; SERODIA^®^-TP-PA, Fujirebio, Tokyo, Japan) at a titer of >1:20,480. A negative RPR and polymerase chain reaction (PCR) for *T. pallidum* and positive TPPA antibodies at a titer of 1:320 in the cerebrospinal fluid (CSF) were detected. The PCR is thoroughly described in Supplementary File S1. 

Other laboratory workup showed an increased sedimentation rate (120 mm/h) and C-reactive protein (34 mg/L), a high platelet count (543 × 10^9^/L) and elevated liver enzymes (aspartate aminotransferase 6.35 µkat/L, alanine aminotransferase 2.19 µkat/L, gamma-glutamyl transferase 4.23 µkat/L). White blood cell and red blood cell counts were normal (5.8 × 10^9^/L and 4.05 × 10^12^/L, respectively).

Urine drug screening was positive for cannabinoids. HIV, and hepatitis B and C virus serology were negative. Fungal and mycobacteria cultivation was negative. In addition to the presence of *S. aureus* in skin swabs, we also detected *T. pallidum* in skin lesions through PCR testing.

#### 2.2.3. Imaging Studies

The MRI angiography of the aorta, ultrasound of the heart, chest X-ray, and CT scan of the head, skull base and paranasal sinuses were normal. Ultrasound of the abdomen showed chronic parenchymal liver damage, and the ultrasound of peripheral lymph nodes showed reactive lymphadenitis, which were most abundant on the neck.

#### 2.2.4. Otorhinolaryngological Check-up

An otorhinolaryngology, ophthalmology, cardiology and infectious diseases specialist examined the patient to stage the burden of the disease on other organ systems. At the otorhinolaryngological check-up, the patient reported no dizziness, vertigo, imbalance, hearing loss, tinnitus or aural pain. Instead, she reported odynophagia and dysphagia with a right-sided foreign body sensation, early-onset hoarseness, and severe pain in the nasal soft tissues, upper lip and teeth. In addition to the previous cutaneous findings (Figure 1A–C), endonasal examination revealed diffuse mucosal oedema, redness, thick and clear nasal discharge, and hyperplastic, red and painful upper gingiva mucosa. In addition, there was a whitish, granulated oval erosion in the right tonsillar region (Figure 1D). An exophytic small lesion was found on the left vocal cord. Vestibular dysfunction and hearing loss were excluded after performing an extensive audiovestibulological workup, which included pure-tone audiometry, caloric vestibular testing, video head impulse test (vHIT), subjective visual vertical (SVV) and cervical evoked myogenic potentials (cVEMP).

These clinical findings indicated the presence of cervicofacial syphilis, comprising extensive skin involvement of the face and neck, gingivitis, syphilitic angina and circumscribed laryngeal lesions.

#### 2.2.5. Histopathology Results of Biopsy Samples

**Figure 2:**
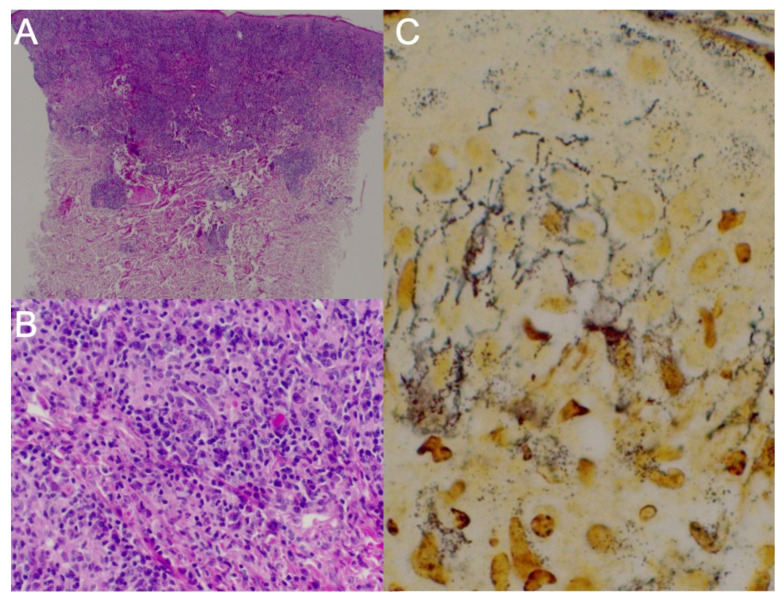
Histopathology analysis of biopsy specimens of a patient with secondary malignant syphilis. (**A**,**B**) Suprascapular skin biopsy sample under low (**A**) and higher (**B**) magnification. Within the superficial and mid dermis is a dense, diffuse inflammatory infiltrate composed mainly of atypical T-cells, numerous plasma cells and histiocytes. (**C**) Facial skin biopsy: Warthin–Starry staining identified spirochetes.

For histopathologic evaluation, two skin punch biopsy specimens and tonsillar region biopsy specimens were obtained. First, the biopsy from the left subscapular region showed an atypical T-cell proliferation with a focal aberrant immunophenotype, expressing monoclonality with rearrangements in the T-cell receptor beta (TCRB) gene, most likely due to infection with *T. pallidum* (Figure 2A,B). A lymphoma could therefore not be ruled out. Another biopsy was thus recommended in the persistent skin lesions after antibiotic treatment.

Histopathological analysis of the right tonsillar region biopsy specimen indicated findings consistent with a syphilitic infection, though nonspecific. Syphilitic involvement could not be confirmed in these lesions because no evident spirochetes were shown by Warthin–Starry staining. We performed immunohistochemical staining for CMV and EBV, for which we did not prove infection, and special periodic acid–Schiff and Grocott staining in which no fungal infection was seen. The histological picture did not show pathognomonic histopathological features of HSV infection such as balloon degeneration cells, intranuclear inclusions and multinucleation. Immunohistochemical staining for HSV was negative.

Studies performed to identify hemolymphoid infiltrates indicated a spectrum of reactive changes.

Lastly, the biopsy performed on the face revealed granulomatous dermatitis caused by *T. pallidum* infection. A direct immunofluorescence test was uncharacteristic. Immunohistochemistry was performed: no Kappa/Lambda restriction; CD20+ in some cells; CD138+ in numerous cells; CD2+/−, CD3+, CD5+, CD7+, CD4/CD8 = 1:3; less than 5% of the cells were CD30+, CD56− and CD34+ in endothelial cells; Epstein–Barr virus + in some cells; and perforin + and T-cell intracytoplasmic antigen+. Spirochetes were identified after Warthin–Starry staining (Figure 2C).

### 2.3. Treatment

The patient was treated for MS, and tertiary syphilis was less likely. Due to TPPA antibodies in the CSF, we decided to treat it as neurosyphilis, although the patient had no neurological manifestations. Otosyphilis and ocular syphilis were also excluded. 

After the alleged penicillin allergy was excluded, the patient received 18 million IU of benzylpenicillin intravenously daily for 14 consecutive days. The treatment was tolerated well by the patient. In addition, she received intravenous corticosteroids before benzylpenicillin to prevent a Jarisch–Herxheimer reaction.

She subsequently became afebrile, we observed healing of the erosions, and the sore throat disappeared. There was a decrease in white blood cell counts to 5.3 × 10^9^/L and platelets to 361 × 10^9^/L, an increase in red blood cell count to 3.35 × 10^12^/L, a decrease of inflammatory parameters and normalization of liver enzymes values.

**Figure 3:**
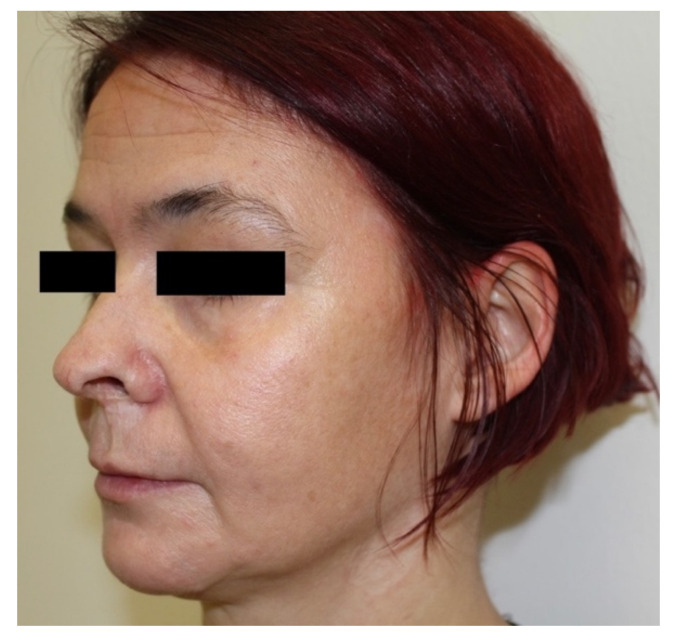
Residual scars on the face and neck 10 months after treatment of malignant syphilis.

At an outpatient check-up 10 months later, residual scars were visible on the skin (Figure 3), and the RPR titer was 1:4. The patient had no functional sequelae of *T. pallidum* infection. The patient attempted suicide and suffered from domestic violence 12 months after the last dermatovenereological check-up.

## 3. Discussion

MS is a very rare disease and only 45 cases have been reported in the literature in the last 5 years [7]. The occurrence of MS rose from 1980 to 2018 [7]. After this period, the incidence plateaued. In 2018, 52 cases of syphilis were diagnosed and reported in Slovenia, and about 10 of them were staged as secondary (according to the 20% ratio of secondary syphilis) [8]. To our best knowledge, this is the first case of MS reported in our country.

As in our patient, MS presents with severe unspecific prodromal systemic manifestations, which can be often attributed to other diseases, and a diagnosis of syphilis is overlooked. After a latent period, disseminated papules occur, which evolve to oval, well-demarcated ulceronecrotic crusted plaques, which can be grotesque and painful, and resemble the gummatous lesions seen in tertiary syphilis [9,10]. Most commonly affected are the skin of the trunk and extremities, but lesions can also erupt elsewhere, even on the mucous membranes [3]. MS can progress to neurosyphilis, otosyphilis and ocular syphilis, which pose an even higher threat to a patient [11,12].

Although TPPA antibodies were positive in CSF, neurosyphilis was excluded after neurological workup and imaging studies. This is consistent with reports that about half of patients with MS and positive CSF antibodies exhibit neurological manifestations [12]. In addition, since otosyphilis can develop at any stage of disease [13], an extensive audiovestibulological workup was performed, which excluded otosyphilis.

The histopathological characteristics of MS can overlap with tertiary syphilis to some degree [9,10]. Histopathology in MS usually shows a dermal infiltrate with lymphocytes and plasma cells and sometimes granulomatous changes [14], as seen in our patient. Although positive immunohistochemistry results and the absence of spirochetes have been reported in MS in the literature [14], immunohistochemistry was not positive in our samples. Warthin–Starry staining was diagnostic for *T. pallidum* infection in our patient’s biopsy samples, consistent with the literature [15].

Interestingly, our case mimicked a cutaneous T-cell lymphoma clinically, histologically and molecularly by expressing monoclonality with rearrangements in the T-cell receptor beta (TCRB) gene. However, plasma cells and spirochetes in subsequent biopsy specimens confirmed secondary syphilis.

Although there are some reports of MS in immunocompetent patients [16], other occult immune deficiencies should be questioned [7]. Our patient reported chronic marijuana abuse and was malnourished, which are known to cause immune suppression [17]. Moreover, she had a mental illness (i.e., attempted suicide). All these predisposing factors could explain the development of a malignant variant of secondary syphilis, as reported in the literature [3,7]. These findings indicate the importance of obtaining a thorough history of psychoactive substance abuse and mental health in syphilis. Despite these facts, it is also known that development of MS can result in more virulent strains of *T. pallidum*.

Our patient’s history was unreliable regarding the origin of the infection, and her known sexual partners screened negative for the infection. However, the patient had been a registered nurse in the past, and that could theoretically explain other less plausible modes of transmission, e.g., via nongenital lesions, human blood or infected objects [18].

## 4. Conclusions

Malignant syphilis is a rare form of secondary syphilis, presenting a grotesque clinical picture and systemic manifestations. It leads to life-threatening complications if left untreated. In a mentally ill patient with chronic drug abuse with nonspecific systemic manifestations and dermatological abnormalities involving the head and neck region, malignant syphilis should be suspected. An extensive multidisciplinary workup, including otorhinolaryngological consultation, guided by a dermatovenerologist is mandatory.

## Supplementary Materials

The following supporting information can be downloaded at: https://www.mdpi.com/article/10.3390/tropicalmed7030047/s1, Supplementary File S1: Molecular detection of *Treponema pallidum*.
