# Supplementary material for: Malignant Syphilis in a Female Patient: A Case Report and Mini-Review
Source: Trop Med Infect Dis. 2022 Mar 8;7(3):47. doi: 10.3390/tropicalmed7030047 (PMC8950934; doi:10.3390/tropicalmed7030047)
Supplement: Supplementary file 1 [file tropicalmed-07-00047-s001.zip › tropicalmed-1600355-Supplementary file-eng-done.pdf]

SUPPLEMENTARY FILE – Molecular detection of *Treponema pallidum*

T. pallidum was mollecularly detected from a sample of the skin lesion, collected with a dry swab (Copan, Brescia, Italy) from cerebrospinal fluid. The swab sample was mechanically pretreated with the MagNA Lyser device (Roche, Berlin, Germany) using MagNA Lyser Green Beads (Roche) and 600 µl of MagNA Pure Bacteria Lysis Buffer (Roche). A volume of 410 µl of swab fluid or cerebrospinal fluid was processed on the MagnaPureCompact instrument (Roche), loaded with a MagNA Pure Compact Nucleic Acid Isolation Kit I (Roche), according to the DNA Bacteria V3 purification protocol. DNA was then eluted in 100 µl of elution buffer.

The LightMix® Modular Treponema pallidum Dual Kit (TIB Molbiol, Berlin, Germany), which targets a specific fragment of the polymerase polA gene and a specific fragment of the 47 kDa lipoprotein gene, was used with the LightCycler Multiplex DNA Master (Roche), according to the manufacturer's instructions. Real-time PCR was performed using the LightCycler 480 II Real-Time PCR System (Roche).
